# Capability of Dielectric Resonator Based Meta-Atoms with VO_2_ Components for Switchable Coding and Wavefront-Manipulating THz Metasurfaces

**DOI:** 10.3390/ma19122449

**Published:** 2026-06-08

**Authors:** Andriy E. Serebryannikov, Kanan Fataliyev, Atilla O. Cakmak, Evrim Colak

**Affiliations:** 1 Division of Physics of Nanostructures, ISQI, Faculty of Physics and Astronomy, Adam Mickiewicz University, 61-614 Poznan, Poland; kanfat@st.amu.edu.pl; 2 College of Engineering, Grand Valley State University, Grand Rapids, MI 49504, USA; cakmaka@gvsu.edu; 3 Department of Electrical Engineering, Ankara University, 06830 Golbasi, Turkey; ecolak@ankara.edu.tr

**Keywords:** phase-change material, vanadium dioxide, meta-atom, metasurface, coding, dielectric resonator, resonance

## Abstract

Vanadium dioxide (VO_2_) is a phase-change material, which changes its properties under thermal or optical stimuli. Thanks to the fact that the material phase transition appears at conditions which are close to environmental ones, VO_2_ has been widely used in diverse structures, including metasurfaces, that acquire switching and reconfigurability capabilities. In this paper, we numerically study the functionality-enabling properties of dielectric resonator-based nondiffractive meta-atoms that comprise small VO_2_ components, i.e., covers or drops, in switchable coding and wavefront-manipulating scenarios at THz frequencies. The goal is to unveil the potential of these meta-atoms in switching the reflected wave’s phase coverage under temperature variations. The main attention is paid to how the shape and size of the VO_2_ components affect the functionality switching that is enabled by the changes in coverage. It is shown that metallic and insulator states of VO_2_ can play different roles in diverse switching scenarios. Different resonance regimes exert different influences on the resulting capability of switching, while contributing to multifunctional operating scenarios. Possible roles of state-dependent absorption are clarified.

## 1. Introduction

Vanadium dioxide (VO_2_) is a PCM, which changes from the amorphous to crystalline phase under thermal or optical stimuli [1,2,3,4,5,6,7]. In a wide frequency range, including the THz range, it corresponds to the transition from the insulator state (i-VO_2_) to the metallic state (m-VO_2_). A significant advantage of VO_2_ is that the transition can occur under conditions that are close to environmental ones. The insulating state can be observed, for instance, when the temperature is *T* = 310 K, while the metallic state does so at *T* = 365 K. The *T*-dependence of the VO_2_ conductivity, σVO_2_, exhibits hysteresis, such that transition occurs at a slightly larger *T*-value during heating. The material phase transition can be obtained for VO_2_ using a conventional heater/cooler.

The above-mentioned features make VO_2_ a promising material for switchable applications in various parts of the electromagnetic spectrum [8,9]. First, we should mention metasurfaces, known also as quasiplanar metamaterials, that are composed of identical (equal) or nonidentical meta-atoms based on dielectric or quasiplanar metallic resonators, or simple nanoantennas [10,11,12,13,14,15,16,17,18,19,20,21,22,23,24]. The achievable switching scenarios are pre-determined by the way in which VO_2_ is incorporated into the meta-atoms. Indeed, it can be used in the form of thin uniform spacer layers or inserts or pads for the resonance components or serve as a material for the entire resonance component. It depends on the available fabrication techniques, on the one hand, and on the targeted functionality, on the other hand.

Other tunable materials, e.g., graphene [25,26], ITO [27], InSb [28,29,30,31,32], and phase-change chalcogenides like GST [33,34,35,36,37], have been successfully utilized in metasurfaces and meta-gratings. In turn, VO_2_ can be incorporated into core–shell scatterers [31,32,38,39,40]. From the metasurface perspective, it is worth mentioning that for the supercell-based approach for wavefront-manipulating metasurfaces, e.g., for deflection or focusing, the functionality is pre-determined by a specific gradual variation in EM wave’s phase from one unit cell (meta-atom) to another [41,42,43,44,45,46,47]. In coding metasurfaces, the phase variation on coordinate(s) can be based on different principles than in the wavefront-manipulating ones [18,48,49,50,51,52,53]. Nevertheless, for both coding and wavefront-manipulating metasurfaces, switching between the operating regimes is possible due to the incorporation of PCM components. Moreover, programmable and intelligent metasurfaces should be mentioned [33,54,55]. Notably, switchable metasurface-based absorbers [36,56,57] and color filters [58] do not need the supercell design, so they may consist of identical (equal) meta-atoms.

In this paper, we numerically study the effects of the material phase of VO_2_ that is incorporated into the meta-atoms based on dielectric resonators for use in reflection mode coding and wavefront-manipulating metasurfaces in the THz range. This frequency range is likely the most promising for exploiting the switching potential of VO_2_. While the circuit-based design solutions, e.g., those using multiple individually controlled diodes, have been shown to be very efficient for switching, mainly, at microwave frequencies [51], they are unlikely to be useful for metasurfaces in the THz range and at higher frequencies. Typically, coding metasurfaces for GHz and THz ranges are based on quasiplanar metallic resonators [48,49,50,51,52,53]. At the same time, dielectric resonators have already been used in THz metasurfaces beyond the coding ones, e.g., see [59,60]. The potential of dielectric resonators in coding metasurfaces for THz range remains unexplored, despite the promise of a rich variety of resonance regimes and a high level of knowledge accumulated for dielectric resonators [42,43,44,45,46]. This work aims to partly fill this gap.

This study is dedicated to dielectric (Mie-type) cylindrical resonators, assuming that the resonances of other types (i.e., not Mie-type) can also contribute to the resulting resonance regimes. Structures with two different thicknesses of small VO_2_ components will be compared. They are realizable by using fabrication techniques consistent with the required structural sizes. The main goal is to unveil the potential of the switchable meta-atoms based on simple dielectric resonators in coding metasurfaces and other metasurfaces that need switchable coverage of the reflected wave’s phase as the basis for functionality switching. The applied mechanism of the phase range switching exploits the changes in the material properties of VO_2_ that are enabled by the transition from i-VO_2_ to m-VO_2_, or vice versa. 

It will be clarified how the thickness/shape of the VO_2_ components may affect the resulting properties of the switchable meta-atoms and related switchable functionalities. Moreover, the role of various resonance regimes in the functionality switching scenarios enabled by variations in *T* will be studied. The role of different resonance regimes will be considered from the multifunctionality perspective, assuming that different functionality switching scenarios can be achieved at different frequencies due to the simultaneous use of two different resonance regimes. It will be demonstrated which functionality switching scenarios can be achieved in the case of dielectric resonators, whereas optimization of the structure design is beyond the scope. The presented results are obtained by using CST Studio Suite 2020 [61].

## 2. Basics

**Figure 1:**
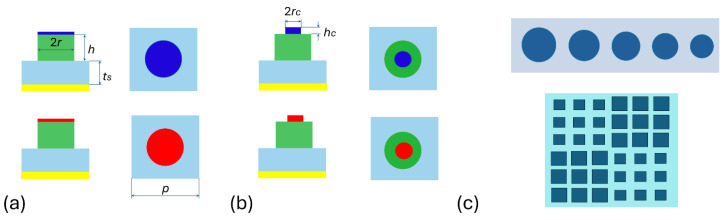
Meta-atoms comprising (**a**) cylindrical dielectric resonators with VO_2_ covers of the same radii as resonators, or (**b**) cylindrical dielectric resonators with VO_2_ covers of smaller radii. (**c**) Exemplified geometries of (**upper plot**) a supercell containing meta-atoms with different cylinder radii and (**lower plot**) fragment of a simple coding metasurface with two different meta-atoms.

The EM wave’s phase coverage that occurs due to the supercell’s meta-atoms with different geometrical parameters and the nearly constant and high magnitude are known as the key characteristic, which determine the capability of coding and wavefront-manipulating metasurfaces that operate in reflected mode [40,41]. The changes in the material phase of VO_2_ is expected to lead to those in phase range coverage and, hence, to the changes in terms of functionality and functionality switching. The key component of the structural design of meta-atoms in this work is a cylindrical dielectric resonator, which can host low-order Mie-type resonances, while intra-array coupling and effects exerted by other resonances also contribute to the resulting regimes. The individual meta-atoms are diffraction-free. The supercells composed of the meta-atoms are diffractive, but they are not the subject of this study. The simplest structural design of meta-atoms is considered. Compared to quasiplanar metallic resonators, dielectric resonators involve the third dimension to allow for hosting more diverse resonances. This can be very crucial for multifunctional/multiband operation. Figure 1 shows the schematics of the studied meta-atoms and exemplified fragments of metasurfaces.

Each meta-atom contains (1) a cylindrical dielectric microresonator made of a high-permittivity material like Si; (2) a VO_2_ cover or drop placed atop the resonator; (3) a spacer made of a low-permittivity dielectric material; and (4) a back-side metallic reflector. Functionality switching scenarios are expected to depend on variations in the radius of the dielectric resonators and the width and thickness of VO_2_ covers/drops. When *T* is varied VO_2_ changes the state from insulator to metallic, or vice versa [1,2,3,4,5,6], the boundary conditions atop the dielectric resonators are also changed; see Figure 1a. In fact, two different sets of resonance modes should correspond to the two cases of effective boundary conditions at the upper resonator surface. Some modes can be weakly affected but the others strongly affected by this change. It is worth noting that the surface control of wave guiding by means of thin covers has been implemented in diverse metastructures [62,63], a larger part of which are not tunable. However, formalization of the effective boundary conditions can be complicated, because of the finite thickness of the conformal VO_2_ cover (*rc* = *r*). The same is true in the case of VO_2_ drops (*rc* < *r*). Moreover, in the i-VO_2_ case, the cover is not expected to function as a perturbation for all modes, and the same can be said about the dielectric resonators having “drops” on top; see Figure 1a,b.

In the studied designs, resonators are asymmetric along the cylinder axis (*z*-axis) owing to the different boundary conditions at their top and bottom. Generally speaking, this asymmetry does not differ from that in most of the dielectric resonator-based metasurfaces, in which resonators are bound by a dielectric substrate or spacer at one side but have air or another host dielectric at the other, e.g., see [42,43,44,45]. At the same time, resonators show infinite-order rotational symmetry in the metasurface plane, i.e., in the (*x*,*y*)-plane. The structure’s symmetry properties result in the absence of cross-polarized components. Notably, metasurfaces with Mie-type resonators comprising VO_2_ components have been studied earlier at the near-infrared and visible ranges [64,65,66].

For the full-scale gradient metasurfaces enabling wavefront manipulation, a proper distribution of the EM wave’s phase along the *x* (and *y*) direction(s) is required. Therefore, the supercells of metasurfaces are composed of different meta-atoms that may have rather closely spaced frequencies, while |S11| keeps nearly the same value, which is aimed to be close to unity. In the case of the deflecting metasurfaces, the EM waves’ phase gradients, *dϕ*/*dx* or/and *dϕ*/*dy*, should be linear, while the whole phase range (*ϕ*-range) extended from 0° to 360° is covered. The phase variation is usually connected with Snell’s law written in the 1D-case in the generalized form [40,41]:

(1) $$n_{t}\sin\alpha_{t}-n_{i}\sin\alpha_{i}=\left(\lambda /2\pi \right)d\phi /dx$$

where *λ* stands for the EM wave’s length; *n_i_* and *n_t_* mean refractive indices in the incidence/reflection and transmission regions, respectively; *a_i_* and *a_t_* represent incidence and refraction angles, respectively. For the purely reflective configuration, *n_t_* in (1) should be substituted by *n_r_* = *n_i_*, so that the phase gradient is still responsible for possible deviations from specular reflection. In the case of the focusing gradient metasurfaces, a parabolic distribution of the phase is needed, which is given in the 1D-case by [40,41]:

(2) $$\phi \;(x)=\frac{2\pi F}{\lambda }-\frac{2\pi {\left(x^{2}+F^{2}\right)}^{\frac{1}{2}}}{\lambda }$$

where *F* stands for the focal length. For the coding metasurfaces [48,49,50,51,52,53], the continuous EM wave’s phase distribution is replaced with discrete phase values, so that each of these values is associated with a particular EM state. From this perspective, even a simple binary phase set may yield a shaped scattering pattern due to the interference of the encoded elements. Phase profiles may be more arbitrary than for the gradient wavefront-manipulating metasurfaces. In the 1-bit coding scheme (0 and 1), two different meta-atoms (known as coding particles) enable binary phase coding, i.e., *ϕ* = 0° and *ϕ* = 180° [50]. In the 2-bit coding scheme (00, 01, 10, and 11), four different meta-atoms are needed, which should correspond to *ϕ* = 0°, *ϕ* = 90°, *ϕ* = 180°, and *ϕ* = 270° [50]. In the 3-bit coding scheme (000, 001, 010, 011, 100, 101, 110, and 111), eight meta-atoms are required to obtain the phase values of 0°, 45°, 90°, 135°, 180°, 225°, 270°, and 315° [50]. The pattern function of the entire coding metasurface under plane–wave illumination can be written as follows [49]:

(3) $$f\left(\beta ,\varphi \right)=\sum_{m=1}^{N}A_{mn}\exp\left[-i\left(m-\frac{1}{2}\right)kD_{x}\sin\beta \cos\varphi \right],$$

where $A_{mn}=\sum_{n=1}^{N}\exp\left[-i\varphi \left(m,n\right)-i\left(n-\frac{1}{2}\right)kD_{y}\sin\beta \sin\varphi \right]$, *β* and φ mean elevation and azimuthal angles, respectively, $D_{x}$ and $D_{y}$ are dimensions of each meta-atom lattice, *k* is the free-space wavenumber, and *N* is the number of lattices over *x* and *y* directions. Figure 1c presents two examples of simple building blocks for coding and wavefront-manipulating metasurfaces.

The energy balance can be introduced as *R* + *A* = 1, where *R* and *A* are reflectance (reflection efficiency) and absorptance, assuming that the back-side reflector’s thickness is chosen so that the transmission is equal to zero. Absorption is enhanced at the resonances, while the extent of possible enhancement needs detailed analysis. Therein, *R* can take (much) smaller values than the minimal acceptable ones. Reflection efficiency can be written as *R* = |S11|^2^, where S11 is the S-parameter responsible for co-polarized reflection [61]. For instance, if the lower limit is set as min|S11| > 0.85, then *R* > 0.72.

At least three idealized scenarios of the change in EM wave’s phase coverage at the transition from i-VO_2_ to m-VO_2_ can be distinguished:

(1) ϕ≈0° for i-VO_2_/m-VO_2_ and ϕ≈360° for m-VO_2_/i-VO_2_.
(2) ϕ≈180° for i-VO_2_/m-VO_2_ and ϕ≈360° for m-VO_2_/i-VO_2_.
(3) Absorption and, hence, no real coverage for one of the m-VO_2_ and i-VO_2_ states and 180° or 360° coverage for the other.

The third scenario may have significant limitations, because A≈1  should be achieved for one of m-VO_2_ and i-VO_2_ states simultaneously at different values of *r* or *r_c_*. However, if absorber functionality is targeted, switchable minimums of *R* may enable a switchable absorber, provided that the metasurface comprises all the same meta-atoms with VO_2_ components. Fortunately, the phase *ϕ* should be discretized in both coding and wavefront-manipulating metasurfaces; this makes minimization of the unwanted effects of narrowband absorption possible.

In line with [11], permittivity of VO_2_ is introduced as $\varepsilon_{VO2}=\varepsilon_{\infty }-\frac{\omega_{p}^{2}}{\left[\omega \left(\omega +i\gamma \right)\right]}$, where $\varepsilon_{\infty }=9$ is high-frequency permittivity, $\omega_{p}=4.988\times 10^{15}\;s^{-1}$ is angular plasma frequency for m-VO_2_, $\omega_{p}=8.346\times 10^{13}\;s^{-1}$ is angular plasma frequency for i-VO_2_, and $\gamma =4.44\times 10^{14}\;s^{-1}$ is collision frequency. The back-side reflector’s material, Cu, has conductivity $\sigma_{Cu}\approx 5.96\times 10^{7}\;Sm^{-1}$. The permittivity of Si is taken as $\varepsilon_{Si}=12.2$, and that of a spacer material as $\varepsilon_{sp}=2.25$. For dielectric cylindrical resonators, a microfabrication approach that combines SU-8 assisted bonding, photolithography, and deep reactive ion etching can be used [59,60]. This fabrication procedure has tolerances in the order ±1 μm for resonator diameter. The VO_2_ layer of nanometer thickness (e.g., 200 nm) can be deposited by using magnetron sputtering [67] before patterning. In the case of micrometer thickness (e.g., 2 microns), the microcrystal-based approach [68] or the nanocrystal-array-based approach [69] is needed. Notably, VO_2_ films being up to 2000 nm thick have been realized earlier by using fabrication steps which can differ from those required for the VO_2_ crystals [70].

## 3. Results and Discussion

Magnitudes and phases of S11 are presented in this section for different parameter sets. The basic features observed in the magnitude results include the minimums, whose spectral locations indicate the presence of resonances. At the sharp minimums of |S11|, strong absorption is achieved. The resonances usually create jumps in the EM wave’s phase to cover either the full or the partial range of phase variation, depending on the demanded functionality. The values of frequencies and radii that correspond to the strong or even moderate minimums should be avoided, except for the designs in which near-unity absorption plays the role of the OFF state in ON/OFF switching scenarios.

### 3.1. Varying Radius of Cylindrical Dielectric Resonators for Thick Conformal VO_2_ Covers

**Figure 2:**
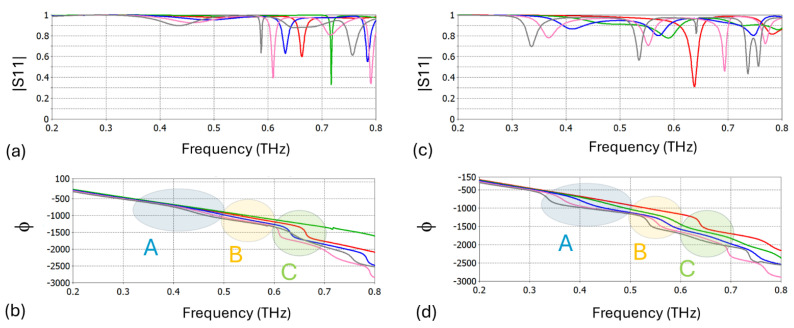
Magnitude (**a**) and phase in degrees (**b**) for S11 in the case of i-VO_2_; magnitude (**c**) and phase in degrees (**d**) for S11 in the case of m-VO_2_ at *h* = 130 μm, *ts* = 150 μm, *h_c_* = 3 μm, and normal incidence (*θ* = 0°) for five values of *r* = *r_c_*: red line—*r* = 50 μm, green—*r* = 60 μm, blue line—*r* = 70 μm, light-rose line—*r* = 80 μm, and gray line—*r* = 90 μm. A, B, and C indicate three ranges which differ in resonance properties and expected functionality.

In Figure 2, the results are presented for the selected values of *r*, which are swept from 50 to 90 μm, while the VO_2_ components fully cover the tops of cylinders. The VO_2_ thickness is taken here as 3 μm, for which the fabrication procedures are available [68,69]. In the case of i-VO_2_, Δ*ϕ* is about 50° in the vicinity of *f* = 0.35 THz for the lowest-*f* resonance range (denoted by A). Therefore, it cannot be used even for 1-bit coding, i.e., the functionality that needs the smallest *Δϕ*-range. However, it is increased up to 180° at *f* = 0.465 THz while min|S11| > 0.92, so it is a candidate for 1-bit coding. Similarly, for the second range (denoted by B), Δ*ϕ* ≈200° is achieved at *f* = 0.57 THz while min|S11| > 0.98. For the third range (denoted by C), Δ*ϕ* >360° is achieved, if using the entire range of *r* variation, for which min|S11| > 0.4. If the case of *r* = 90 μm is excluded, then min|S11| > 0.6 at 0.61< *f* < 0.67 THz. Therefore, range C can be potentially applicable to 2-bit and 3-bit coding and wavefront-manipulation functionalities, provided that the deep minimums of |S11| are avoided due to the properly selected discrete values of *r*. Higher resonances arising at *f* > 0.7 THz can also be utilized.

Spectral location and manifestations of resonance regimes can be changed when *T* is increased and the metallic state of VO_2_ is achieved, because the effective boundary conditions at the upper resonator surface are changed; see Figure 2c,d. For the resonance range denoted by A, Δ*ϕ* from 250° to 310° is achieved. Notably, Δ*ϕ* > 250° is kept in a wide band, i.e., at 0.35 < *f* < 0.50 THz. This range can be a candidate for 2-bit coding, offering the bandwidth which is about 35%. For the range denoted by B, Δ*ϕ* > 360° at *f* = 0.57 THz, where min|S11| > 0.8, so it can be used for 3-bit coding. When *r* is swept only from 50 to 70 mm, |S11| > 0.85 and Δ*ϕ* ≈200°. Finally, for the range C, Δ*ϕ* can be close to 360° while min|S11| > 0.8 at 0.6 < *f* < 0.68 THz. It corresponds to the 12.5% bandwidth, but it can be difficult to realize it because of the minimums of *A*. Sweeping over *r* from 60 to 90 μm leads to Δ*ϕ* = 290° at *f* = 0.663 THz, whereas min|S11| > 0.94. Involvement of *r* = 50 μm allows us to increase Δ*ϕ* up to 360°. The two above-mentioned ranges, i.e., 0.35 < *f* < 0.50 THz and 0.6 < *f* < 0.68 THz, can be considered as the wideband operation ranges, wherein Δ*ϕ* is weakly or gradually variable. The obtained results show that both states of VO_2_ may yield the coverage of the entire phase range or a required part of it, but the metallic state suggests more options. Typically, the resonance regimes leading to larger bandwidths create worse phase coverage but suffer less from enhanced absorption. 

Design strategy can be based on the choice of metallic state operating frequencies, and the double checking of scenarios arising at these frequencies for i-VO_2_. Table 1 presents a few examples. Evaluation of their applicability for coding and wavefront manipulation can be carried out based on the results obtained for Δ*ϕ* and min|S11|.

**Figure 3:**
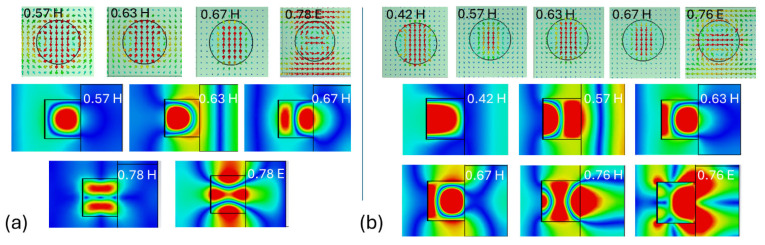
A few examples of the field distribution for (**a**) i-VO_2_ and (**b**) m-VO_2_, at the same geometric parameters as in Figure 2. In each plot, the frequency value is given in THz (0.42, 0.57, etc.); letters H and E denote magnetic and electric fields, respectively. Vector maps are presented for the selected cross-sections over the cylinder height. Color maps correspond to the mid-cross-section of the cylinder); EM wave is incident from the left side.

Figure 3 presents the examples of field distribution for two *f*-values taken from Table 1 (0.42 and 0.57 THz) and other resonance regimes observed in Figure 2. The contribution of magnetic-dipole resonance is obvious in most of the presented field plots. However, its functionality-enabling properties that are connected with magnitude and phase of S11 may differ because (1) the fields created by different resonances can be localized within different (and poorly predictable) regions in the same resonator, so that the field localization region can be shifted towards the upper or the lower part of the cylinder, depending on *f*; (2) fields created by two different resonances can be simultaneously present, occupying different regions. The effect of changing VO_2_ state is observed in Figure 3 at *f* = 0.57, 0.63, and 0.67 THz. It leads not only to the field changes near the cover, but also within the subregions which are quite far from the cover. The largest region for magnetic-dipole resonance has been observed at *f* = 0.57 THz and 0.6 THz for the i-VO_2_ and *f* = 0.63 THz and 0.67 THz for the m-VO_2_. The magnetic-dipole resonance is expected to yield the jumps of *ϕ* at *f* = 0.63 THz in the i-VO_2_ case and at *f* = 0.42 and 0.57 THz in the m-VO_2_ case, while the spacer resonances may also contribute to the resulting resonance regimes.

In the case of i-VO_2_, the electric-dipole resonance starts to contribute at *f* = 0.62 THz, when the field only occupies the region that is close to the spacer. This region gradually extends with increasing *f*, so the effect of the electric-dipole is observed over the entire height of the cylinder, e.g., at 0.76 and 0.78 THz. Electric-dipole resonance is responsible for the phase jump of *ϕ* that appears near 0.78 THz (see Figure 2), but the effect of magnetic resonance becomes significant at 0.79 THz (it is localized in the lower part of the cylinder). In the case of m-VO_2_, contribution of the electric-dipole resonance was observed starting from *f* = 0.7 THz, when it occupies the part of the cylinder that is closest to the spacer. Similarly to the i-VO_2_ case, the occupied region is increased with *f*, achieving the maximal volume at *f* > 0.75 THz. Electric-dipole resonance is expected to be responsible for the phase jump near 0.76 THz, whereas the effect of magnetic resonance is observed in the region adjacent to the cover.

In addition, effects of vertical electric dipoles (in contrast with horizontal dipoles in Figure 3), or more advanced resonances, can be expected at 0.6 THz for m-VO_2_ and at 0.67 THz for i-VO_2_, but it requires a deeper study. Intra-array coupling may also contribute to the resulting resonance features. Notably, proper use of multipole decomposition and other techniques can be complicated due to the ambiguity of choosing the effective distance from the spacer, possible contribution of spacer resonances, and a way of formalization of intra-array coupling.

**Figure 4:**
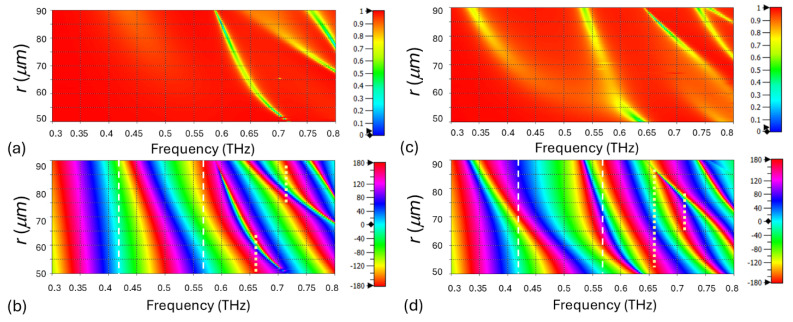
Magnitude (**a**) and phase in degrees (**b**) for S11 on (*f*,*r*)-plane in the case of i-VO_2_; magnitude (**c**) and phase in degrees (**d**) for S11 on (*f*,*r*)-plane in the case of m-VO_2_, at *h* = 130 μm, *ts* = 150 μm, *rc* = *r*, *h_c_* = 3 μm, and normal incidence. Dashed white lines indicate two cases taken from Table 1. Dotted yellow lines indicate the ranges of *r* possible for covering the full range of *ϕ* for the selected values of *f*.

For the design purposes, presentation of results on magnitude and phase results on the frequency–geometric parameter plane can be advantageous. Figure 4 presents the results when *r* is gradually swept from 50 to 90 μm, while the remaining parameters are kept the same as in Figure 2. To use these results for the entire 2π-range coverage, two criteria should be satisfied simultaneously:

(1) In the phase plots, it should be possible to pass from red to red through all other colors, at a given *f* and VO_2_ state; it determines the range of *r* variation which is potentially applicable to cover the entire range of *ϕ*; accordingly, a pass from red to cyan is needed for the 180° coverage.
(2) In the magnitude plots, the chosen *r*-ranges and *f*-values should be checked, i.e., whether the condition |S11| > S_min_ is satisfied, where S_min_ is taken, for instance, as 0.8.

To estimate the capability of the selected range of *r* at chosen value of *f* in switching, it is necessary to compare magnitude and phase results for the m-VO_2_ and i-VO_2_ cases in Figure 4. An important advantage of the use of the (*f*,*r*)-plane is that the required range of *r* variation can be directly evaluated for each value of *f*, for which desired phase and, hence, color changes are achieved. For instance, it can be used when the entire phase range should be covered, i.e., Δ*ϕ* = 2π for one of the VO_2_ states, while it is either small (no more than several tens of degrees) or close to π for the other. As observed, sharper resonances may yield a narrower range of the required variation in *r*. It is recommended to select wider ranges of *r* (i.e., the ones for which the colors are changed slowly while *r* is varied), since fabrication imperfections are expected to be within ±1 μm for the cylinder diameter [59,60]. Two examples of the potentially applicable range of *r* are shown by dashed yellow lines for each of the two VO_2_ states.

Precise discretization of the *ϕ*-profile is crucial if narrow resonances, like the resonances in range C in Figure 2b,d, are involved. However, since the required number of discrete values of *ϕ* is typically not larger than eight, it should not be an unsolvable problem. The other situation may occur if a larger number of discrete values of *ϕ* is needed. A noticeable feature is that max*A* can be achieved at rather arbitrary values of *r*, for both cases of i-VO_2_ and m-VO_2_ [see the small blue and green regions in Figure 4a,c]. For instance, one of the regimes of A≈1 is achieved for i-VO_2_ when f≈0.6 THz and *r* = 85 μm. The slopes of the “valleys” of max*A* are different for different resonance regimes, which are pre-determined by the specifics of the field distribution.

### 3.2. Varying Radii of Cylindrical Dielectric Resonators for Thin Conformal VO_2_ Covers

**Figure 5:**
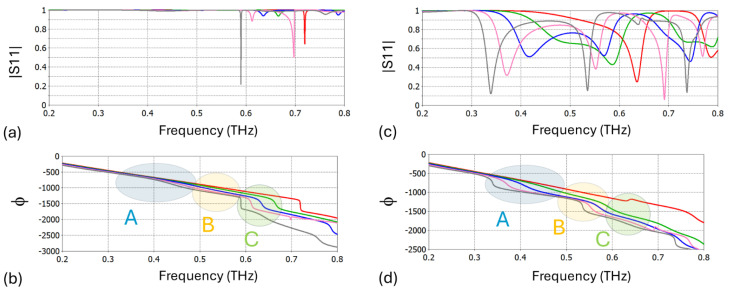
Magnitude (**a**) and phase in degrees (**b**) for S11 in the case of i-VO_2_; magnitude (**c**) and phase in degrees (**d**) for S11 in the case of m-VO_2_ at *h* = 130 μm, *ts* = 150 μm, *h*_c_ = 300 nm and angle of incidence *θ* = 0°, for five values of *r* = *r*_c_; red line—*r* = 50 μm, green—*r* = 60 μm, blue line—*r* = 70 μm, light-rose line—*r* = 80 μm, and gray line—*r* = 90 μm. A, B, and C indicate three ranges which differ in resonance properties and expected functionality.

To compare with the results from Section 3.1, Figure 5 presents |S11| and *ϕ* vs. *f* for the case of *hc* = 300 nm. Such a thickness of VO_2_ is widely used in tunable and switchable metasurfaces. Sputtering [67] or chemical vapor deposition [70] can be used to obtain a thickness of this order. Three typical frequency ranges can be distinguished depending on the behavior of |S11| and *ϕ*. Similarly to the case of *hc* = 3 μm, they are denoted by A, B, and C. For the case of i-VO_2_, typical values of *ϕ* are, respectively, tens to 180, 200, and 360 degrees. In range C, the use of *r* which varies from 50 μm to 80 μm can be sufficient to cover the entire phase range. For the two remaining ranges, the use of the whole range of *r*, i.e., from 50 μm to 90 μm, can also be superfluous. The presented results indicate that the spectral locations of the resonances for the two used values of *hc* can be close to each other but their widths and depths can differ. As follows from the comparison of the results in Figure 2 and Figure 5, not only the 3 μm thick covers but also 300 nm thick ones may enable various ranges of *ϕ* coverage in the i-VO_2_ state, while absorption depends on the thickness. Indeed, deeper maximums are observed for some values of *r* when *hc* is smaller, but shallower maximums appear for the others. The impedance matching technique can be used to explain the appearance of the absorption maximums [71], which correspond to the reflection-free regime. 

Also, in the case of m-VO_2_, the features observed in Figure 5 look like the ones in Figure 2 but typically show stronger minimums of |S11|. This makes the bands for possible wideband operation narrower and the overall design more complicated, because it becomes more difficult to overcome the ranges of strong absorption when discrete values of *r* are selected. Notably, absorption is generally not proportional to the volume of the absorbing material (e.g., see [71]), so it is not surprising that the minimums of |S11| in Figure 5c are deeper than in Figure 2c. This may occur due to the specific phase and impedance conditions leading to such a field localization which results in higher absorptance at smaller *hc*. 

If a strong absorption regime is planned to be incorporated into the switching scenario as the OFF state, i.e., in line with scenario 3 listed in Section 2, then *hc* = 300 nm may be preferable. However, it is better suited for equal meta-atom metasurfaces, but not for supercell-based metasurfaces. It can be said that the larger thickness of VO_2_ components can provide some protection against stronger absorption at 0.3 < *f* < 0.6 THz. It is worth noting that the approach based on the results of the (*f*,*r*)-plane, like in Figure 4, can also be used for thin covers. Table 2 presents a few examples of the phase range coverage that is associated with different functionalities and different functionality switching scenarios. The range 0.35 < *f* < 0.465 THz can be considered as a band suitable for 2-bit coding, which has a bandwidth of 28%, but the switching scenario depends on the choice of *f*-value. Note that the fact that min(Ω_Δϕ_, 1/Ω_Δϕ_) is of the order of unity at *f* > 0.465 THz (see Table 2) does not mean that this range cannot be used in the switching scenarios, but values of *f* and *r* should be carefully selected in this case.

**Figure 6:**
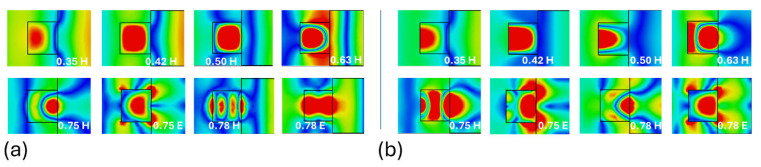
A few examples of the field distribution for (**a**) i-VO_2_ and (**b**) m-VO_2_, at the same geometric parameters as in Figure 5. In each plot, the frequency value is given in THz (0.35, 0.42, etc.); letters H and E denote magnetic and electric fields, respectively. Color maps correspond to the mid-cross-section (side view); EM wave is incident from the left side.

Examples of field distributions corresponding to the selected spectral regimes from Figure 5 and Table 2 are presented in Figure 6 for *r* = 70 μm. From the comparison of Figure 6a,b, effects exerted on the field by the change in VO_2_ state are evident. Similarly to the case of *hc* = 3 mm in Figure 3, a magnetic resonance is dominant at the frequencies, where larger values of Ω_Δϕ_ can be obtained. For i-VO_2_, it occurs, for instance, at *f* = 0.63 THz. For m-VO_2_, it happens at *f* = 0.42 and 0.63 THz. Also, the effects related to intra-array coupling and spacer resonances can be significant. The electric-dipole resonance comes into play at higher frequencies. For i-VO_2_ and m-VO_2_, its effect starts to appear at least from 0.66 THz and 0.75 THz, respectively. At other values of *r* like 50 μm and 90 μm, sharper resonances may occur in range C and close to it [see Figure 5b].

### 3.3. Effect of Spacer Thickness

**Figure 7:**
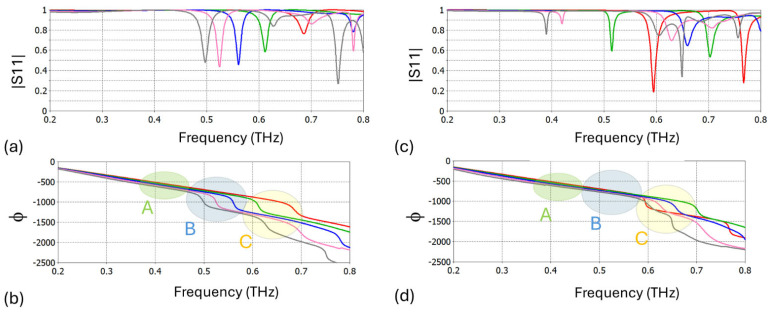
Magnitude (**a**) and phase in degrees (**b**) for S11 in case of i-VO_2_; magnitude (**c**) and phase in degrees (**d**) for S11 in case of m-VO_2_, at *h* = 130 μm, *ts* = 50 μm, *h_c_* = 3 μm, *θ* = 0°, for five values of *r* = *r_c_*: red line—*r* = 50 μm, green—*r* = 60 μm, blue line—*r* = 70 μm, light-rose line—*r* = 80 μm, and gray line—*r* = 90 μm. A, B, and C indicate three ranges which differ in their resonance properties.

The spacer thickness is another important parameter that may affect metasurface performance. The results are presented in this section for values of *ts* that can be chosen. Clearly, *ts* should be the same for all meta-atoms in each supercell. Figure 7 presents the results for |S11| and *ϕ* for five selected values of *r*, when *ts* is chosen as 50 μm. These results show that |S11| and *ϕ* still strongly depend on *r*, while the effect exerted by *ts* is significant. Three typical ranges, A, B, and C, are distinguishable. It is worth comparing two ranges, B and C. For range C, a strong dependence of the spectral locations of min|S11| on *r* occurs for the both cases of m-VO_2_ and i-VO_2_, but it looks more regular and better predictable for m-VO_2_. In turn, range B looks more suitable for ON/OFF switchable scenarios because well-pronounced resonances that are strongly sensitive to the variations in *r* appear only for i-VO_2_. As a result, we can obtain here a higher contrast between two states of VO_2_. Finally, no useful switching regimes can be obtained for range A, where Δ*f* only achieves several tens of degrees for both i-VO_2_ and m-VO_2_. Notably, not all of the minimums of |S11| yield the jumps of *ϕ*. For i-VO_2_, the first jump that may enable Δ*f* = 360° is spectrally shifted from 0.5 to 0.68 THz, while *r* is increased from 50 up to 90 μm. It remains true for m-VO_2_ starting from *r* = 60 μm, whereas the jump’s spectral location for *r* = 50 μm nearly coincides with that for *r* = 90 mm. As a result, the well-pronounced phase jumps start to appear at 0.6 THz for m-VO_2_, instead of 0.5 THz for i-VO_2_. Note that the results presented may significantly differ not only from those in Figure 2 but also from the ones for other values of *ts*.

**Figure 8:**
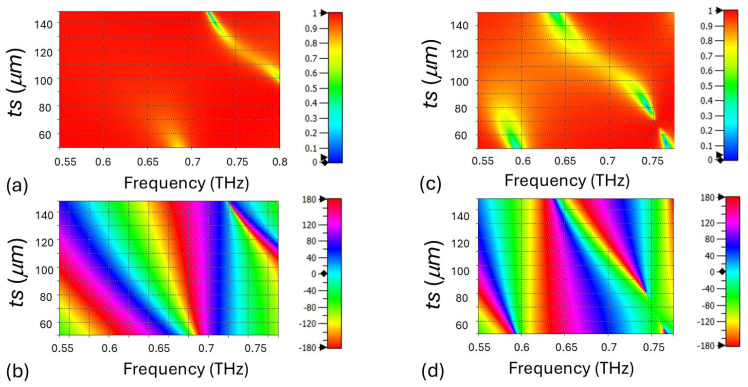
Magnitude (**a**) and phase in degrees (**b**) for S11 on (*f*,*ts*)-plane in the case of i-VO_2_; magnitude (**c**) and phase in degrees (**d**) for S11 on (*f*,*ts*)-plane in the case of m-VO_2_; *h* = 130 μm, *r* = *rc* = 50 μm, *hc* = 3 μm, and *θ* = 0°.

Figure 8 presents the results on the (*f*,*ts*)-plane, i.e., in a similar manner like in Figure 4. The radius *r* = 50 μm is chosen here. The small dark-blue regions in Figure 8a,c correspond to the case of A≈1, and should be avoided in designs of supercell-based metasurfaces. Clearly, such regions appear for m-VO_2_ and i-VO_2_ at different *f* and *ts*. Note that there are *ϕ* ranges in which the phase weakly depends on *ts*, while strong sensitivity to the variations in *ts* occurs for the others. Moreover, the features associated with BIC [72] are recognizable in both magnitude and phase plots. For instance, it happens for m-VO_2_ [see Figure 8d] in the vicinity *f* = 0.75 THz when *ts* = 70 μm. The presence of BIC may affect the possible choice of *ts*, which will be studied in detail in the next steps of this research program. 

### 3.4. Varying Size of Non-Conformal VO_2_ Drops/Covers

Besides variations in the sizes of dielectric microresonators, there is one more way to design different phases of EM waves and, hence, different meta-atoms in one supercell. It can be done by means of size variations in the VO_2_ components, i.e., drops or covers. At the micrometer scale, it might be possible to place these components atop the resonators in a controlled way. It can be difficult to provide eight different meta-atoms (with different radii of *rc*) in one supercell or in the entire structure. However, using two different values of *rc* in switchable 1-bit coding or four for switchable 2-bit coding may look realistic.

**Figure 9:**
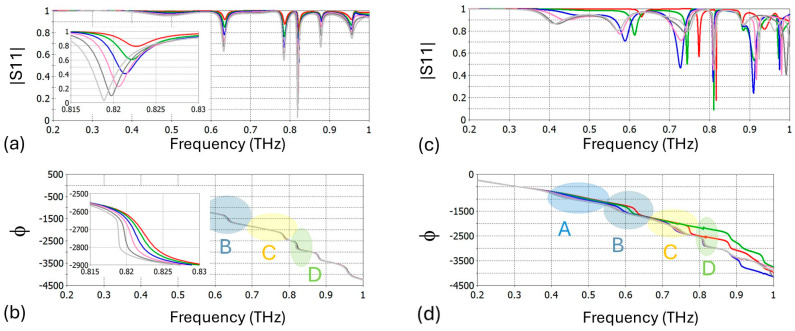
Magnitude (**a**) and phase in degrees (**b**) for S11 in the case of i-VO_2_; magnitude (**c**) and phase in degrees (**d**) for S11 in the case of m-VO_2_ at *r* = 70 μm, *h* = 130 μm, *ts* = 150 μm, *hc* = 3 μm, and *θ* = 0°, for five values of *rc*: red line—*rc* = 20 μm, green—*rc* = 30 μm, blue line—*rc* = 40 μm, light-rose line—*rc* = 50 μm, gray line—*rc* = 60 μm, and light-gray line—*rc* = 70 μm. A, B, C, and D indicate four ranges which differ in resonance properties and expected functionality.

Figure 9 presents the magnitude and phase of S11 vs. *f* for five meta-atoms, which differ in the value of *rc*, while *tc* = 3 μm is kept constant. Both |S11| and *ϕ* remain without significant change beyond the resonance regions, but they can differ near the resonances so that the resonance frequencies and EM wave’s phase can be slightly different at different values of *rc*. In the case of i-VO_2_, the difference is significant for the resonance observed at *f* = 0.82 THz (within the range denoted by D), at which Δ*ϕ* > 200°. At the same time, Δ*ϕ* does not exceed a few tens of degrees for the lower-*f* ranges. Due to this feature, they are expected to be suitable rather for the OFF state, in line with scenario 3 from Section 2. The magnitude plot clearly shows the deep minimums for the resonances arising in range D, which contribute simultaneously to the phase range coverage and high absorption. Hence, they are also usable in the OFF state. Absorption strongly depends on *rc*, whereas the spectral shift in resonances is relatively small. This regime can be used in metasurfaces comprising all the same meta-atoms, as applicable for switchable absorption, at least if the resonance is shifted while changing from i-VO_2_ to m-VO_2_.

In the case of m-VO_2_, the results are similar, in some sense, to the results presented in Figure 2 for the meta-atoms using the same VO_2_ state. In Figure 9, Δ*ϕ* = 200° is achieved for range A and Δ*ϕ* = 300° for range B. The range 0.42 < *f* < 0.56 THz constitutes a band with a width of 28.5%, in which |S11| is kept without significant changes. A careful adjustment of the values of *rc* is needed in the m-VO_2_ case to avoid significant absorption at the minimums of |S11|. As said above, the resulting absorption is not proportional, in the general case, to the volume occupied by VO_2_, which is typical for absorbers of different types; for example, see ref. [71]. Furthermore, spectral locations of the resonances in ranges A and B are sensitive to the variations in *rc*, even if small portions of VO_2_ are used. On the other hand, just a weak dependence of spectral locations of resonances on *rc* is observed in ranges C and D. For instance, resonances in range D are just slightly shifted when i-VO_2_ is changed for m-VO_2_, or vice versa. In many cases, the difference between strong sensitivity in the m-VO_2_ case and weak sensitivity in the i-VO_2_ case can be more pronounced when variations in *r* are substituted by the ones in *rc*.

**Figure 10:**
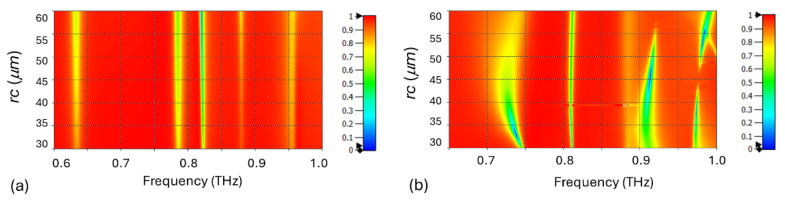
Magnitude of S11 on (*f*,*rc*)-plane in the case of (**a**) i-VO_2_ and in the case of (**b**) m-VO_2_, when *rc* is varied from 30 to 60 μm, *h* = 130 μm, *ts* = 150 mm, *r* = 70 μm, *hc* = 3 μm, and *θ* = 0°.

Next, Figure 10 shows |S11| on the (*f*,*rc*)-plane. The results are presented for the case when *hc* = 3 μm, *rc* is varied, while the remaining parameters are the same as in Figure 9. Similarly to Figure 4, these results can be directly used for design purposes, i.e., for supercells containing meta-atoms with different *rc*. The key feature observed in the case of i-VO_2_ is that absorption is monotonously increased at resonances with *rc*. However, for m-VO_2_, the minimums of |S11| are observed at intermediate values of *rc*. Therefore, the optimal values of *rc* can be found for narrowband absorption with A≈1.

**Figure 11:**
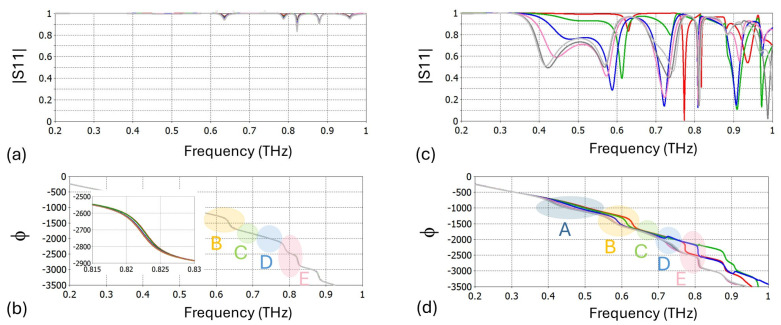
Magnitude (**a**) and phase in degrees (**b**) for S11 in the case of i-VO_2_; magnitude (**c**) and phase in degrees (**d**) for S11 in the case of m-VO_2_ at *r* = 70 μm, *h* = 130 μm, *ts* = 150 μm, *hc* = 300 nm, and *θ* = 0°, for five values of *rc*: red line—*rc* = 20 μm, green—*rc* = 30 μm, blue line—*rc* = 40 μm, light-rose line—*rc* = 50 μm, gray line—*rc* = 60 μm, and light-gray line—*rc* = 70 μm. A, B, C, D, and E indicate five ranges which differ in resonance properties and expected functionality.

To compare, Figure 11 presents the results for the case when *hc* = 300 nm, and the remaining parameters are the same as in Figure 9. A weak sensitivity to the applied variations in *rc* is observed in the case of i-VO_2_. In other words, the covers/drops work as weak perturbations that do not affect the EM wave’s phase, whereas min|S11| > 0.82 is kept for all values of *rc* within the entire *f*-range. Therefore, it can serve as an OFF state (corresponding to specular reflection), as in scenario 3 in Section 2. In the case of m-VO_2_, the observed behavior of *ϕ* vs. *f* is similar to Figure 2 and Figure 5, at least for ranges A and B. Accordingly, Δ*ϕ* that is close to 200° and 350° can be achieved. 

The results obtained in the case of m-VO_2_ confirm that even thin and small VO_2_ components may exert a strong effect on |S11| and *ϕ*. However, absorption can be undesirably high, as observed for the range extended from 0.4 to 0.6 THz; see Figure 11c,d. This can be a serious restriction for use in coding metasurfaces. Nevertheless, it is still possible to select the case with min|S11| > 0.7, which occurs at *f* = 0.5 THz, for operation in equal meta-atom metasurfaces. Supercell metasurfaces can be designed, but with a relatively low efficiency, in which m-VO_2_ serves as the ON state for 1-bit coding, while i-VO_2_ is responsible for the OFF state. At the same time, narrow resonances in ranges C and D correspond to a nearly perfect absorption and can be used for ON/OFF switchable absorption. Hence, at least two switchable scenarios are still possible in the case of *rc* < *r*, i.e., for switchable 1-bit coding and switchable narrowband absorption. Finally, the possibility of using range E remains under question. 

## 4. Concluding Remarks

The performed study has unveiled the effects exerted by variations in geometric parameters of meta-atoms comprising dielectric microcylinders and small VO_2_ components. It is shown that a variety of switching scenarios is possible even for a simple design that is based on the dielectric resonators of the cylindrical shape. The basic differences between the cases of m-VO_2_ and i-VO_2_ were compared in terms of coverage of the reflected wave’s phase range and related functionalities. As expected, functionality-enabling resonances can be controlled by varying boundary conditions atop the dielectric resonators while applying heating or cooling. It enables switchable coverage of the EM wave’s phase that is necessary in switchable coding functionalities. The sensitivity to the transition of VO_2_ from the insulating to the metallic state depends on the chosen resonance regime that may yield diverse switching scenarios, which serves as a prerequisite for switchable multifunctional operation. Typical scenarios include switching between 1-bit coding and 3-bit coding, and between ON/OFF switching of 2-bit coding. While magnetic resonances are the key enablers of the desired functionality, their joint effect with other resonance effects may enforce the performance.

The microcyliner radius is the basic parameter whose variation allows for obtaining different phases for supercell-based designs. In this case, the achievable functionalities and functionality switching scenarios may depend on the thickness of the conformal VO_2_ cover in such a way that may look unexpected at first glance. A larger thickness of VO_2_ can be preferable, since it allows for reducing absorption within the frequency and geometric parameter ranges, which are suitable for coding and wavefront manipulation at THz frequencies. 

Varying the size and shape of the VO_2_ components at a given size of dielectric resonators formally gives one more degree of freedom for obtaining the switchable phase coverage. Similarly to the case when cylinder radiu*s* is varied, the phase range coverage differs from one resonance regime to another, when the neighboring meta-atoms have the VO_2_ covers of different radii. The most interesting feature in this case is that the strong sensitivity of the EM wave’s phase and spectral locations of resonances to the variations in cover/drop radius may occur in the m-VO_2_ case, while the same variations only lead to insignificant changes in the EM wave’s phase in the i-VO_2_ case. The results show that the functionality switching scenarios can be obtained by using small non-conformal VO_2_ components. For larger thicknesses of VO_2_*,* larger diversity of functionality switching scenarios can be obtained, while efficiency is a weaker restriction than in the case of smaller thicknesses. Different resonance regimes and the specifics of their modification under temperature variations correspond to different functionality switching scenarios. Usually, electric- and magnetic-dipole resonances are considered as the basic resonances in dielectric cylinders. However, spacer resonances and intra-array coupling may also contribute to the resulting functionality. 

In turn, a proper choice of spacer thickness can help to avoid strong absorption for all discrete values of the radius of the cylinder or cover that are required within one supercell for phase coverage. In realistic cases, fabrication imperfections should just slightly affect the coverage ranges. However, sharp resonances are more sensitive and, hence, are worth overcoming. The unwanted parameter sets can be detected by using the frequency –geometric parameter plane or geometric–parameter plane at a given frequency for reflected wave magnitude and phase. In fact, this way of presenting the results is very efficient regardless of which geometric parameter is varied. So, it can be recommended for a wide class of metasurfaces based on subwavelength resonators. At oblique incidence, two linear polarizations can be used for coding independently of each other. It should be noted that when the EW wave’s phases are well adjusted for one of the VO_2_ states, they can be just approximately adjustable for the other. Therefore, a device-oriented design procedure can be more complicated than the presented one, whose aim was to retrieve basic features and provide suitable entry-point information for future (pre-)prototype designs. At the same time, the results obtained provide proper guidelines for next-stage studies of subwavelength meta-atoms.

Comparison of the capability of subwavelength dielectric and quasiplanar metallic resonators in functionality-enabling switchable scenarios will be a subject of future studies. The concept of switchable functionality, which depends on the chosen resonance regime, can be replicated in other frequency ranges, assuming that different sizes and, likely, other tunable materials are utilized. Moreover, it is planned to be examined for possible use in devices exploiting magneto-optical effects and spin waves.

## Figures and Tables

**Table 1:** Examples of phase range coverage and expected switching scenarios in reflection mode in the case of thick conformal covers at *f* < 0.62 THz; Ω_Δϕ_ = Δϕ_met_/Δϕ_ins_ is the phase coverage contrast.

f (THz)	Δϕ_ins_	Δϕ_met_	Ω_Δϕ_	min|S11|_ins_	min|S11|_met_	Switching Scenario
0.334	53°	183°	3.45	0.992	0.75	imperfect specular reflection–1-bit
0.35	51°	295°	5.78	0.987	0.865	imperfect specular reflection–2-bit
0.42	100°	295°	2.95	0.907	0.87	imperfect specular reflection–2-bit
0.57	205°	>360°	>1.7	0.979	0.80	1-bit–3-bit
0.60	215°	>360°	>1.7	0.933	0.81	1-bit–3-bit

**Table 2:** Examples of phase range coverage and expected switching scenarios in reflection mode at the selected frequencies in case of thin conformal covers at *f* < 0.63 THz; Ω_Δϕ_ = Δϕ_met_/Δϕ_ins_ is the phase coverage contrast.

f (THz)	Δϕ_ins_	Δϕ_met_	Ω_Δϕ_	min|S11|_ins_	min|S11|_met_	Switching Scenario
0.35	49°	300°	6.1	0.997	0.46	imperfect specular reflector–2 bit
0.465	180°	264°	1.45	0.92	0.712	1 bit–2 bit
0.50	200°	230°	>1	0.994	0.65	only magnitude switching
0.62	>360° with or without *r* = 50 μm	220° without *r* = 50 μm	<1	0.98	0.55	3 bit–1 bit
0.66	>360°	264° without *r* = 50 μm	<1	0.973	0.813	3 bit–2 bit

## Data Availability

The original contributions presented in this study are included in the article. Further inquiries can be directed to the corresponding author.

## Abbreviations

The following abbreviations are used in this manuscript:

PCM	phase-change material
BIC	bound states in the continuum
EM	electromagnetic
